# A feasibility study evaluating the usability and acceptability of the personalised space technology exercise platform mobile application

**DOI:** 10.1038/s41598-025-20314-0

**Published:** 2025-10-17

**Authors:** Hannah M. Worboys, Laura J. Gray, Sarah Anthony, Rachel Hobson, Tim Lucas, Joshua D. Vande Hey, Marios Panagi, G. André Ng

**Affiliations:** 1 Department of Population Health Sciences, University of Leicester, Leicester, UK; 2 Department of Cardiovascular Sciences, University of Leicester, Leicester, UK; 3 Earth Observation Science Group, School of Physics and Astronomy, University of Leicester, Leicester, UK; 4 Center for Environmental Health and Sustainability, University of Leicester, Leicester, UK; 5 Climate and Atmosphere Research Center, The Cyprus Institute, Aglandjia, Cyprus; 6 Department of CardiovascularSciences, Leicester BHF Centre of ResearchExcellence, Leicester NIHR Biomedical Research Centre, University of Leicester, Leicester, UK

**Keywords:** Environmental sciences, Health care, Medical research, Risk factors, Signs and symptoms

## Abstract

The P-STEP (Personalised Space Technology Exercise Platform) app is designed to bring together tailored exercise guidance and up-to-date air quality information for individuals with long term health conditions. The app allows individuals to plan outdoor exercise walking routes while minimising their exposure to air pollution. Individuals with chronic long-term conditions, particularly respiratory and cardiovascular conditions, can use the app to minimise the risk of their symptoms being worsened by pollution while gaining the benefits of outdoor exercise. This study measured the usability and acceptability of the P-STEP app. The study was a single-arm 12-week pilot study based in Leicestershire, United Kingdom. We recruited 93 participants from an existing cohort study to use and evaluate the app for 12 weeks. Questionnaire data were collected at three timepoints; baseline, 6-weeks and 12-weeks. The primary outcome was the System Usability Scale at 12 weeks. Secondary outcomes included the User Engagement Scale Short Form, SF-12, Recent Physical Activity Questionnaire, bespoke app-specific usability questions and feasibility outcomes. Additional data collected include participant demographic information, technology self-efficacy, and adverse events. Weekly anonymised app usage data were collected and analysed separately to complement the questionnaire data. We assessed 342 individuals for eligibility, of whom 182 (53%) were eligible. Of the 182 eligible, 93 (51%) were enrolled and given access to the app for 12 weeks. Overall, 61 (66%) participants completed the study. At 12 weeks, the mean (sd) System Usability Score was 61.68 (22.9), bespoke usability score was 66.82 (14.75) and User Engagement Score was 3.08 (0.79). Completion rates across all questionnaires were high. Participants accepted the format of online questionnaires, with no participants requiring help to complete and no participants withdrawing from the study for this reason. This study helps to understand the feasibility and acceptability of administering the P-STEP app in a cohort of people with relevant long-term conditions. The results will help inform the design of a future randomised controlled trial.

*Trial registration* The https://clinicaltrials.gov/ ID number is NCT05830318.

**Supplementary Information:**

The online version contains supplementary material available at 10.1038/s41598-025-20314-0.

## Introduction

As of 2021, seven million people in the UK live with either a cardiovascular or respiratory long term health condition^1^. These conditions are associated with poor health outcomes, reduced quality of life, increased number of hospitalisations, and premature death. Effective management through medication and lifestyle changes, particularly increased physical activity, can reduce the risk of poor outcomes. Regular physical activity offers many benefits to an individual, including improved physical and mental health, cardiovascular outcomes and survival. Engaging in regular physical activity can also enhance cardiovascular fitness, which helps prevent the progression of long-term conditions (LTCs).

Physical activity guidelines for the general population recommend at least 150 min of moderate-intensity exercise per week^2^, and often similar recommendations exist for those living with chronic conditions^3,4^. Those living with LTCs receive the same benefits from exercise as those without^5–7^. Despite the guidelines, many individuals with LTCs fail to meet current recommended activity levels^8^. Exercise activities are often prescribed for individuals with respiratory and cardiovascular conditions for the specific purpose of mitigating the exacerbation of symptoms related to their disease. This may be, for example, exercise to improve cardiovascular health in heart failure patients, or reduce BMI and increase glucose control in people with type 2 diabetes. Those living with LTCs may be limited in their ability to engage with physical activities due to their LTC, and many may be under medical supervision before and during engagement of physical activity.

One important barrier to physical activity among individuals with LTCs is the risk of exposure to air pollution during outdoor exercise, such as walking, cycling, or running. Physical activity increases ventilation (breathing) rates, leading to higher inhaled doses of pollutants such as particle matter (PM_2.5_), nitrogen dioxide (NO_2_), ozone, and ultrafine particles^9–11^ This can exacerbate respiratory symptoms and contribute to reduced lung function, particularly among individuals with asthma and COPD^12–14^. Negative cardiovascular effects have also been reported, including altered vascular function, blood pressure, and cardiac arrhythmias^15–17^.

Carefully controlled physical activity is possible and can be beneficial in people living with LTCs^18,19^. Mobile phone applications (apps) can be used to support behavioural change in those with LTCs, such as to aid increasing physical activity levels^20^. As well as being in control of LTC symptoms and flare ups, another important factor to those living with LTC is being able to assess the air quality in the local area prior to outdoor exercising^21^. In addition to direct physiological effects, individuals with LTCs may experience additional behavioural barriers due to fear or concern regarding outdoor exposure to pollutants. For example, thunderstorm asthma is an example of a condition influenced by extrinsic environmental exposures. Other extrinsic environmental factors include meteorological factors such as high pollution levels, extreme temperatures, and aeroallergens^22,23^. Pollen exposure more broadly can act as a trigger for respiratory symptoms and exercise-induced bronchoconstriction, particularly in individuals with asthma or allergic conditions. Thunderstorm asthma represents a more acute form of this interaction, where meteorological conditions and high pollen levels combine to trigger severe exacerbations^24,25^. Air pollution might influence people’s attitudes and behaviours towards exercise. Evidence suggests that people may reduce or avoid physical activity during episodes of high air pollution, and air quality alerts may significantly influence people’s decisions to exercise outdoors^26^. In addition to the physical risk of such extrinsic exposures, the fear of potentially encountering these factors might dissuade people with LTCs from engaging in physical activity, making it imperative to be aware and forewarned of these extrinsic conditions potentially occurring.

The Personalised Space Technology Exercise Platform (P-STEP) smartphone app was designed to address this gap by integrating real-time (hourly) air quality data with personalised exercise guidance, allowing individuals to plan safer outdoor activities. The P-STEP app, developed with funding from the European Space Agency, brings together tailored exercise guidance, taking into account an individual’s LTC, while also providing up-to-date information on air quality. The air quality information captured by the app includes a variety of emissions that impact health and activity through short (hours to days) or long-term (months to years) exposure. Air pollution is a complex mixture of gases and particles originating from a wide range of human activities such as vehicle emissions, industrial processes, residential heating, and power generation, as well as natural sources including wildfires, volcanic activity, sea spray, and desert dust^27^. Once emitted, these pollutants can be transported across large distances, with meteorological factors such as wind speed, direction, boundary layer height, and temperature strongly influencing the dispersion and accumulation of particles^28,29^. The P-STEP app provides real-time data on several pollutants of particular relevance to health. Particulate Matter (PM) is monitored in two fractions: PM₁₀ (particles with a diameter smaller than 10 μm) and PM₂.₅ (particles smaller than 2.5 μm). PM₁₀ includes larger particles such as dust, pollen, and sea salt, which primarily affect the upper airways and may aggravate respiratory conditions. In contrast, PM₂.₅ consists of finer particles from combustion sources and secondary chemical reactions, which can penetrate deep into the lungs and bloodstream, and is strongly associated with cardiovascular disease, respiratory illness, and increased mortality^30–32^ Nitrogen dioxide (NO₂) is emitted primarily from fossil fuel combustion in the transport sector, energy production, and residential heating. NO₂ contributes to the formation of secondary pollutants such as ozone and nitrate aerosols and is associated with airway inflammation, impaired lung function, cardiovascular disease, and increased incidence of asthma, especially in children^33,34^. Ozone (O₃) is a secondary pollutant formed through photochemical reactions involving nitrogen oxides (NOₓ) and volatile organic compounds (VOCs) in the presence of sunlight^35^ Exposure to ozone has been associated with lung inflammation, airway hyper-responsiveness and adverse effects on both the cardiovascular and respiratory systems^36,37^.

Air quality data within the P-STEP app is provided through EarthSense’s MappAir model (https://www.earthsense.co.uk/mappair), which combines ground measurements, satellite data, emissions, and meteorological inputs to produce high-resolution, real-time air quality information at 100 m resolution in the United Kingdom and 10 m in the Leicester. Data are updated hourly to give users accurate, location-specific pollution levels.The P-STEP app will access the user’s location (with permission) using satellites around Earth Global Navigation Satellite System (GNSS) in order to help them plan when and where to exercise. The app aims to advise users on where in the local area they can exercise in the cleanest areas possible. P-STEP aids in this goal by helping users to find the cleanest areas local to them and giving them access to a 72-hour forecast to find the optimum time and place to exercise. Long-term studies suggest there could be benefits of regular exercise even at moderate air pollution levels^38,39^.

The app allows individuals to plan exercise routes (walking routes) in order to minimise their exposure to air pollution, by using the information to help them avoid higher polluted areas. Alerts were integrated into the app to bring the user to attention high levels of pollution in the local area. The user was also able to look at the 72-hour forecast in a specified location, allowing them to plan their activities around times where their local area was highly polluted. The app collects data on the time individuals have spent exercising, to reassess their exercise targets which are calculated by a bespoke evidence-based algorithm designed by a team of health experts. This algorithm takes into account the individuals characteristics, LTC, and previous levels of exercise. The user can access a range of features in the app, including recording their walking route, planning when/where to go walking by being provided with a 72-hour forecast, achieve walking badges/milestones, save their favourite walking routes. For such an application to be successful in real-world settings, it must be both usable and acceptable. People with different LTCs might be affected in different ways by different pollutants, therefore filters and personalisation exist to ensure the app is adaptable for the individual.

This feasibility study is crucial as it evaluates the practical implementation of the P-STEP app. By assessing usability engagement and feasibility outcomes, this study will inform the refinement of the app and the design of a future clinical trial. Understanding the barriers and facilitators to technology-driven behaviour change will help optimize future digital health interventions, ensuring their accessibility, relevance, and long-term survival while also improving health outcomes for individuals.

## Methods

### Overview

This feasibility study was a single arm 12-week pilot study based in Leicestershire, United Kingdom (UK). Questionnaire data were collected at three timepoints, baseline, six weeks and 12 weeks. Weekly anonymised usage data from the app were also collected. The South West Frenchay Research Ethics Committee (REC ref: 23/SW/0060) approved the study, confirming that no further Health Research Authority (HRA) approval was necessary. This study has been reported according to the CONSORT 2010 checklist for pilot or feasibility trials^40^. The SPIRIT figure is included in the supplementary information (Figure S1). The protocol for this study has been published elsewhere^41^.

### Recruitment methods and procedure

A combination of purposive and convenience sampling were employed. Purposive sampling due to the specific characteristics required to meet eligibility criteria, and convenience as we recruited from a pre-existing cohort study based in Leicester, United Kingdom. Participants were recruited from the EXCEED cohort study^42^. The EXCEED study (REC ref 13/EM/0226) is a longitudinal population-based cohort study that facilitates the investigation of genetic, environmental and lifestyle-related determinants of a broad range of diseases and multiple long-term conditions^43^. EXCEED participants have consented to be contacted about participating in other research studies. Recruitment was in two stages, first the criteria to meet the initial EXCEED email call out, whereby participants who met the first stage of the criteria (Table 1) were emailed by a member of the EXCEED research team informing them about the study, providing them a copy of the participant information sheet and a link to the P-STEP website. Those interested in registering for the study could fill in a registration form (via Microsoft Forms) with questions asking the second stage of inclusion criteria, detailed below in Table 1. The Microsoft forms were then reviewed by a member of the P-STEP study team. The Microsoft forms had questions regarding the second stage of criteria, then additional criteria to be enrolled on the study. No renumeration was offered for participation in this study, however reimbursements were made if mobile data allowance was exceeded or the user incurred extra charges as a result of participating.

**Table 1:** Inclusion criteria for P-STEP study.

First stage of inclusion criteria: Part of the EXCEED cohort study Adult ≥ 18 years Lives in Leicestershire Does not have dementia, learning disability, severe mental health disorders (other than depression or anxiety), cancer or epilepsy. Are not receiving palliative care Diagnosed with at least one of the following conditions; asthma, chronic obstructive pulmonary disease (COPD), interstitial lung disease (ILD), coronary heart disease (CHD), heart failure (HF), type 2 diabetes* * It was agreed that given the short recruitment window, this criterion could be relaxed to include participants without LTCs if recruitment was slow.
Second stage of inclusion criteria: Does Can walk for a minimum of 5 min outside Have an Android smartphone Have access to the internet on smartphone Have ability to give informed consent Does not Have chest pain at rest Feel unsteady when standing or walking, which has led to a fall Are pregnant Are a current cancer patient Are receiving palliative care Have access to an iOS smartphone only Have been advised not to take part in exercise in the past 12 months Were part of the P-STEP User engagement group and provided PPI input.

### Data collection methods and procedure

Participants who met the inclusion criteria were invited to complete an electronic consent form via REDCap and enrolled onto the study. Participants then filled in the baseline questionnaire and were given access to the P-STEP app. All participants were provided with an onboarding guidance document which included screenshots and instructions of how to download the app, create an account and log in for the first time. When users had logged in for the first time there was an in-app tutorial to familiarise them with the app. The baseline questionnaire asked demographic questions, about current experience using technology, the Recent Physical Activity Questionnaire (RPAQ) and the SF-12 quality of life questionnaire. Each participant had access to the app for 12 weeks. Follow-up questionnaires were sent via REDCap at 6- and 12-weeks. The follow-up questionnaires included the System Usability Scale (SUS), bespoke usability questions, the User Engagement Scale Short Form, RPAQ, SF-12, information about non-routine GP visits or unexpected hospitalisations and general usage and feedback questions about the app. Two free-text questions allowed for collection of additional feedback data. The questionnaires were designed through a process of iteration with our Patient and Public Involvement and Engagement (PPIE) members. Questionnaire data was collected in REDCap and the quantitative data analysed in Stata. Free text responses were summarised.

### Outcomes

#### System usability scale (SUS)

The primary outcome measure for this study was the system usability score (SUS) of the P-STEP app after 12 weeks of use. The SUS is a validated and popular instrument for measuring perceived usability^44^. There are 10 items in total, 5 with a positive tone and 5 with a negative tone. The responses range from strongly disagree to strongly agree. For scoring, the negative SUS responses are reversed and the scores were then transformed onto a 0-100 scale. A SUS score of 68 and above considers the usability score to be above average and anything less below average^44^.

#### Bespoke usability questions (P-STEP specific)

Nine additional usability questions (Table S2) that relate specifically to the features of the P-STEP app, were asked at 6 and 12 weeks. These questions were formulated with input from the PPIE group, and through a process of iteration, finalised with nine questions on a Likert scale of strongly agree to strongly disagree.

#### User engagement scale short form (UES-SF)

The User Engagement Scale - Short Form (UES-SF) contains twelve items that measure user engagement^45^. There are 12 items which are categorised into; focused attention, perceived usability, aesthetic appeal, and reward. Focused attention refers to feeling absorbed in the interaction and losing track of time. Perceived usability refers to the experience of the interaction and the degree of control and effort expended. Aesthetic appeal refers to the attractiveness and visual appeal of the interface. Reward is the extent to which the experience was rewarding. An overall user engagement was calculated by converting the score to a numeric value of 1–5 (strongly disagree equals 1, strongly agree equals 5) and taking the mean^45^.

### Quality of life

We used the SF-12 in this study to give an indication of change and assess the feasibility of the questionnaire in this sample. The Short Form 12-Item Health Survey (SF-12) is a health-related quality of life tool that measures functional health and well-being from the participant’s perspective^46^. It assesses eight domains including physical functioning, physical role, pain, general health, vitality, social functioning, social role and mental health. The 8 domains are then categorised into two summary scores; physical component score (PCS) and mental component score (MCS). Scores for the PCS and MCS range from 0 to 100, and a higher score indicates a higher quality of life.

### Recent physical activity questionnaire (RPAQ)

The RPAQ was used and enables assessment of the daily physical activity and sedentary behaviours of adults^47^. It includes questions on physical leisure and sports activities (frequency and duration), and activities performed in the home (television, computer, climbing stairs, etc.) and at work (quantity and type of work, home-work journeys, etc.). The analysis takes into account the duration and frequency of each activity, and its intensity. We used the RPAQ in this study to give an indication of change and assess the feasibility of the questionnaire in this sample. As well as the RPAQ, two additional questions on self-perceived walking pace were collected at 6 and 12 weeks.

### Feasibility outcomes

Anonymous usage data were extracted weekly and provided information on the features used within the app. The data extracted are a summary of all participants the information is anonymous and therefore cannot be linked back to participants. This data informed the feasibility outcomes which assessed the feasibility of using certain study design features and outcome measures which will help plan a future trial for effectiveness. One of the main feasibility outcomes is the acceptability of the questionnaires within this sample group.

### Statistical analysis

Baseline characteristics are summarised using mean and standard deviation (SD) or median and interquartile range (IQR) for continuous variables and count and percentage/proportion for categorical. The mean and standard deviation of the SUS, usability and user engagement scores is reported at 6 and 12 weeks. A breakdown of responses to the SUS questionnaire are included both as a frequency table and bar graph. The SF-12 and RPAQ were collected at baseline, 6 and 12 weeks. An indication of changes over time are reported using a fixed effects model. Feasibility outcomes are reported as mean and standard deviations or count and percentages where appropriate.

### Subgroup analyses

We conducted three subgroup analyses for the usability outcomes based on (i) whether participants had one of the LTCs or not, (ii) whether participants had experience using a fitness tracking device, and (iii) removing users who enrolled but reported not using the app.

### Objective app usage data

Walking data were exported from the app to completement the questionnaire data. Input from health professionals provided guidance on the thresholds to remove outliers. Outliers were removed under the assumptions that:

- Participants may have forgotten to pause and got into a moving vehicle and as a result, the distance for these walks would be high while the associated steps low.
- Participants may have forgotten to pause when getting back from a walk and as a result, the duration for these walks would be high while the associated distance and steps low.

### Non-routine GP visits and unexpected hospitalisations

As the app is not an investigational medicinal product nor a medical device, no adverse effects are expected. Data on non-routine GP visits and unexpected hospitalisations, related or unrelated to the participant’s chronic condition(s) were collected at 6 and 12 weeks, instead of adverse events. Any unexpected hospitalisations reported were treated as a potential serious adverse event (SAE) as per University of Leicester Sponsor SAE reporting policy, and followed up directly with participants to obtain further information.

### Patient and public involvement and engagement (PPIE)

Members from the University Hospitals of Leicester NHS Trust Lifestyle PPIE group, and members from the Extended Cohort for E-health, Environment and DNA (EXCEED) study were involved in the design of this study. This included designing and reviewing the study documents such as the register your interest form, participant information sheet, informed consent form, baseline and follow-up questionnaires. PPIE members were also included in reviewing the participant pathway to identify ways to minimise participant burden.

## Results

### Recruitment and enrolment

**Fig. 1:**
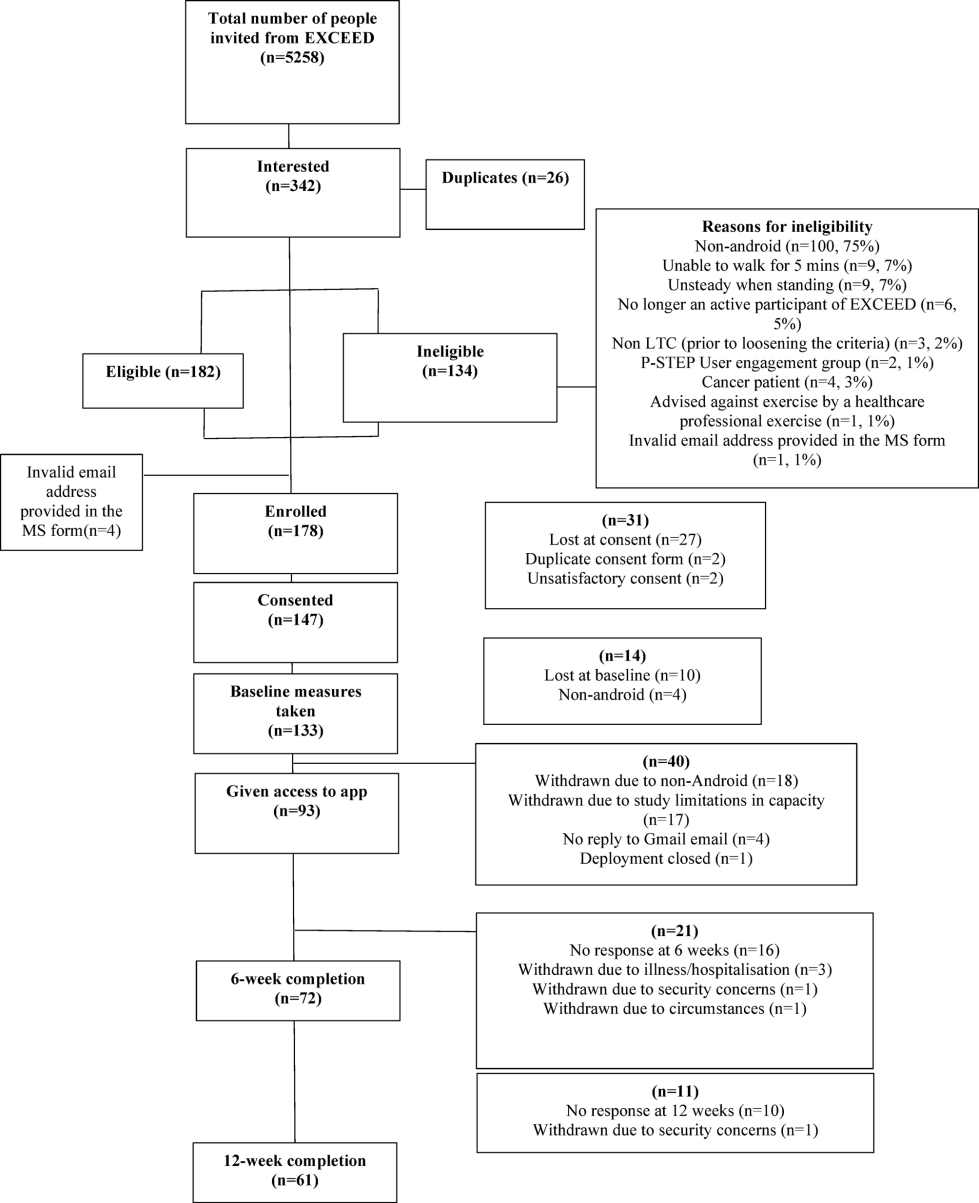
CONSORT flow diagram.

Recruitment to the study was open from August – October 2023. Recruitment was reviewed every two weeks by the study team and mitigation strategies were employed if the study team identified recruitment to be slower than expected. Mitigation strategies for recruitment included relaxing the inclusion criteria of a participant being diagnosed with one of the six long-term conditions. This is justified as while the app may be designed for those with cardiovascular or respiratory diseases, everyone can and would benefit from a reduction in exposure to air pollution. Another justification for this is that the primary outcome is the usability of the app and not its effectiveness in these condition groups. Logistically this mitigation strategy means the EXCEED research team would send out a second wave of invitations irrespective of long-term condition as long as all the other inclusion and exclusion criteria were met. The CONSORT flow diagram for the participants in this study is reported in Fig. 1. Of the 342 people who registered their interest in the study, 182 (53%) were eligible. All but 4 of the 182 were enrolled on the study, however 85 were lost at some point between enrolment and being given access to the app. Of these, 43 (51%) did not respond to emails moving them onto the next stage and 21 (25%) were non-Android users. A total of 17 participants had to be withdrawn from the study due to a delay in getting the app verified by Google.

### Baseline, demographics and technology self-efficacy

Overall, 93 participants were given access to the app and all were included in the final analysis. The baseline demographics of the participants are included in Table 2. Of the final sample, 33 (35%) were diagnosed with one of the 6 long term conditions originally part of the inclusion criteria. A further breakdown for the conditions is included in Table 2. Previous experience and current usage of technology are recorded in Table S1. All participants have experience using a smartphone at least once a week, with the majority (98%) using every day. Most (80%) participants have regular use of a computer and experience using weather apps (85%). However, none reported experience using pollution tracking apps. A third (35%) of participants have experience using health apps and half (49%) reported experience using exercise tracking apps.

**Table 2:** Baseline characteristics experience.

Variable	Mean	Std. dev	Min	Max
Age	66.25	8	48	79

	Freq	%		
Sex, Female	59	63.4		
Ethnicity				
White	83	89		
Mixed/multiple ethnic groups	1	1		
Asian/Asian British	6	6		
Black/African/Caribbean/Black British	3	3		
Prefer not to say	0	0		
Other	0	0		
Long term condition(s)				
Asthma	18	19		
Type 2 diabetes	13	14		
Chronic obstructive pulmonary disease	4	4		
Interstitial lung disease	1	1		
Coronary heart disease	2	2		
Heart failure	1	1		
At least one LTC	33	35.5		
English first language	89	95.7		
Has previously attended an exercise rehabilitation programme	3	3		

### Usability and acceptability of the app

#### System usability scale

Table 3 reports a mean (SD) SUS at 12 weeks of 61.62 (22.09). 34 (56%) reported a SUS score of less than 68, representing somewhat below average usability. Itemised responses at 12 weeks from the SUS are included in Table S2, 50% of participants agreed they would use the app frequently, found it easy to use, 64% felt confident using the app, 66% of participants agreed “most people would learn to use the app quickly”, 76% disagreed additional assistance would be needed to use the app, and 57% disagreed it was awkward to use.

#### Additional usability and user engagement

Table 3 reports a mean (SD) overall score at 12 weeks of the bespoke usability questions of 66.82 (14.75), out of a possible 100. These results indicate a good usability in the user specific functions of the P-STEP app. Itemised responses at 12 weeks are included in Table S2, 82% agreed the app provided them with up-to-date air quality information, 56% felt it was suitable for their age group, 61% agreed the app allowed them to track their own progress, 52% felt reassured the app would keep their data secure, 33% agreed the app provided guidance specific to their needs, 38% disagreed that the app allowed them to plan their walking routes, and 39 agreed the walking guidance recommended matches their walking ability.

Table 3 reports a mean (SD) overall score from the UES-SF of 3.08 (0.79) out of a possible 5, indicating avergae4 user engagement. Itemised responses at 12 weeks are included in Table S2, 54% disagreed they felt absorbed or lost their selves in the experience of using P-STEP, 60% did not find P-STEP confusing to use, 69% did not find it physically or mentally demanding, 56%, 54% neither agreed or disagreed P-STEP was an attractive app or visually appealing, and 61% agreed they found the app interesting.

**Table 3:** Outcome scores.

	Baseline, mean (SD) (*n* = 93)	6 weeks, mean (SD) (*n* = 72)	12 weeks, mean (SD) (*n* = 61)	Effect size mean change over time (95% CI)	*p*
System usability score		57.92 (20.12)	61.68 (22.09)		
Bespoke usability questions		65.62 (15.14)	66.815 (14.75)		
UES-SF overall		3.06 (0.80)	3.083 (0.79)		
Focused attention		2.57 (0.89)	2.519 (0.96)		
Perceived usability		3.37 (0.97)	3.55 (0.99)		
Aesthetic appeal		3.06 (0.84)	3.077 (0.85)		
Reward		3.23 (1.10)	3.224 (1.03)		
SF-12 physical component score	49.57 (6.79)	50.30 (6.51)	49.53 (7.50)	− 0.066 (− 0.656 to 0.523)	0.826
SF-12 mental component score	54.18 (6.23)	53.72 (5.89)	54.03 (6.47)	− 0.433 (− 0.978 to 0.113)	0.120
Sedentary (RPAQ)	6.56 (2.80)	6.29 (2.76)	6.39 (2.50)	− 0.075 (− 0.331 to 0.182)	0.568
Light (RPAQ)	1.08 (1.40)	0.81 (1.32)	0.687 (1.46)	− 0.117 (− 0.220 − 0.014)	0.027
Moderate (RPAQ)	4.96 (1.92)	5.56 (1.61)	3.88 (1.56)	− 0.430 (− 0.639 to 0.222)	< 0.001
Vigorous (RPAQ)	1.02 (0.22)	0.88 (0.23)	0.72 (0.23)	− 0.136 (− 0.156 to 0.117)	< 0.001
Completeness of questionnaires					
SUS	–	99.86%	100%		
Bespoke	–	100%	99.84%		
UES-SF	–	100%	99.84%		
SF-12	99.73%	99.31%	96.45%		
RPAQ	99.33%	93%	88.28%		

			Freq (%)		
Change in perceived walking pace			22 (24%)		

### SF-12

There was no indication of change in quality of life over the 12-week period (Table 3). The SF-12 had a very high completion rate across all 3 time periods (Table 3).

### RPAQ

There was no indication of change in the sedentary domain, while light, moderate and vigorous domains all indicated a negative change in physical activity over the 12 weeks (Table 3).

#### Self-perceived walking Pace

At 12 weeks, 24% of participants agreed that their walking pace had positively changed as a result of using P-STEP (Table S2).

#### Usage and feedback

**Fig. 2:**
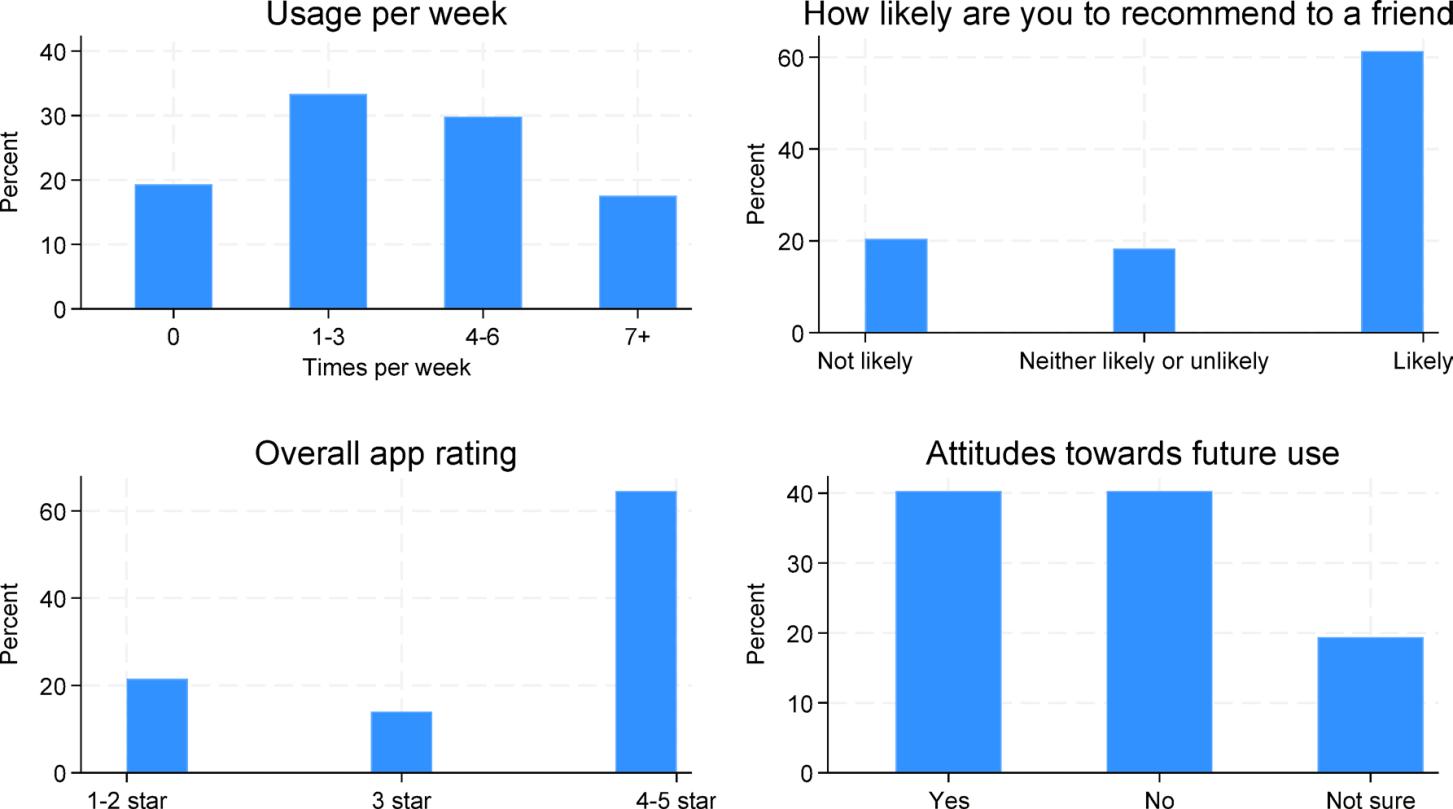
Usage and feedback questions.

Figure 2 presents graphically responses on usage and feedback of the app. Participants were asked on average how many times they used the app per week. 80% reported using the app at least once every week, almost 20% reported using the app every day. 60% of participants reported they would be likely to recommend the app to a friend or family member, and 60% rated the app 4–5 stars.

#### Feasibility outcomes

The original invitation from the EXCEED study team went to 5,258 individuals, this resulted in 342 (6.5%) responses proportion of interested individuals who met the eligibility criteria was 53%, and reasons for ineligibility are recorded in Fig. 1. 98% of eligible participants enrolled on the study. We offered participants the opportunity to receive a phone call to assist with downloading the app and registering for an account, 91% of participants declined this offer and downloaded the app without assistance. 61 (66%) of participants completed the 12-week study. Completion rates across all outcomes were high (Table 3). Participants accepted the format of online registration, informed consent, and questionnaires, no participants needed assistance to complete the online questionnaires and no participants who withdrew from the study recorded this as a reason.

#### Objective app usage data

The total number of walks recorded in the pilot was 2,154. After the removal of outliers, where data either had little to no distance over a large time period, or little to no time period over a large distance, the total number of walks recorded over the 12-week period is 1,557.

#### Free-text analysis

Participants were asked “Please enter any suggestions to improve the app?” and “Please enter any other feedback you have.” Direct quotes are included in Table S5. The responses have been categorised into two themes; the functionality of the app and the concept of the app.

Responses relating to the functionality of the app were passed on to the app design team for consideration in future iterations of the app.

Overall summary of functionality:

- There should be a quick start button to start recording a walk, rather than three taps.
- Feedback questions after the walks should be shorter, more appealing.
- Some of the design featured can be simplified.
- More guidance for some screens.
- People forget to finish and sometimes start their walks. They would prefer an automatic start and automatic pause button. There needs to be a differentiation between moving and elapsed time.
- The amount of information on the screen seemed to be off-putting for some.
- Some mentioned to have received too many notifications.
- Some users requested suggestions for outdoor walks.

Responses relating to the concept of the app were reviewed by the P-STEP study team and will be taken into consideration for future studies relating to the app.

#### Non-routine GP visits and unexpected hospitalisations

Non routine GP visits and unexpected hospitalisations are recorded in Table S3. Of the one unexpected hospitalisation reported, this was reported to Sponsor as a potential SAE, but later withdrawn following clarification from the participant that this was an outpatient visit.

#### Subgroup analyses

The results from the 3 subgroup analyses are recorded in Table S4. When looking at just the participants with a long-term condition (*n* = 33), at 6 weeks, the SUS was 56.67 which was lower than the non-diseased group (58.45). At 12 weeks, the SUS was considerably lower in the diseased group at 56.91 compared with 63.52 in the non-diseased group. When looking at participants with experience of using a fitness tracking device (*n* = 22), at 6 weeks, the SUS was 56.70 which was lower than the non-experienced group (58.47). At 12 weeks, the SUS was 61.05 for experienced and 61.96 for the non-experienced group. We removed 4 users who reported on the questionnaire that they never used the app, and looked at differences between this and if they were included. At 6 weeks, the SUS was 59.40, slightly higher than the original group (57.92). At 12 weeks, the SUS was 62.41, again slightly higher than the original group (61.69). These differences were similar for the bespoke usability questionnaire and the UES. All differences were non-significant.

## Discussion

### Main findings

P-STEP is a unique mobile phone application that combines air quality data with outdoor walking guidance and has the ability to personalise recommendations based on the participant’s LTC. The purpose of this study was to assess the usability and acceptability of the P-STEP app among participants with LTCs. This feasibility study offers valuable insights into the usability and engagement potential of the P-STEP app.

The findings indicate a moderate level of usability and mixed engagement experiences, with substantial feedback on areas for app improvement. While these results are encouraging, several important considerations must be addressed for future iterations and for the design of a future evaluation. The feasibility of administering the P-STEP app was assessed by participants taking part in a 12-week study. This study has provided useful information to assist in the design of a future randomised controlled trial. With regard to the usability, the mean system usability score at 12 weeks equated to a usability score of just below average. Further usability was evaluated from the bespoke usability questionnaires where participants were asked about the specific design features of the P-STEP app. This feedback was more positive and equated to an above average usability score. With regards to acceptability, all domains of the User Engagement Scale equated to medium engagement. Participants provided written feedback relating to the functionality of the app which will be used to improve future iterations of the app. Other studies have shown that PA apps often suffer from similar engagement drop-off issues, especially in older adults or populations with complex health needs^48^. However, some apps that integrate behaviour change techniques (e.g. goal setting, automatic tracking, social support) have shown more promising results^49^.

### Strengths

The completion rates of the questionnaires were high in this sample, with very few participants skipping any questions. The acceptability of online registration, informed consent and questionnaires was high, we were not requested to assist any participants in their completion of informed consent, baseline or follow up questionnaires in REDCap. We recorded no problems directly emailing participants from REDcap to receive questionnaires and reminders. Almost everyone (98%) who registered their interest and was eligible to take part in the study was enrolled. The 4 participants that were not enrolled was due to incorrect email address given, and the study team were unable to make contact. The dropout rate of 33% which is lower than previously reported app feasibility studies^50^. Recruitment through the EXCEED study provided us with access to a variety of participants in terms of health status and experience using technology. This allowed us to pilot the app in a varied group, giving us more nuanced feedback and differing perspectives, which will help further with the future development of the app and design of a randomised controlled trial.

### Limitations

This study did not appeal greatly to individuals in this cohort, with a modest response rate of 6.5% (*n* = 342/5258). There are a number of possible reasons for this. Firstly, there may be a reluctance in this cohort to take part in app studies due to a lack of confidence in using technology and security concerns around testing an app in the investigational stages of development. The time of year of the study may have also affected take up. The study ran from September to January, where the weather and daylight timings may have discouraged individuals to take part in the study with the expectation of needing to use the app to walk outdoors.

Despite the study team emphasising the fact this was an Android only study, 18 participants made it through to the final stage of being given access to the app before realising they have a non-Android phone. As a research team we reflected on this and concluded:

- What we thought may have been a thorough description of an Android smartphone may not have been, though this specific issue was not picked up on in the PPI meetings or reported in feedback forms.
- Participants may be unaware of the type of phone that they have.
- Extra checks in future work would need to be employed to ensure this issue is addressed in the early stages and does not waste the time of the participants or research team.
- Highlights the need for apps to be developed on both smartphone platforms (IOS and Android).

Due to the Android restriction, 130 people registered their interest in the study who couldn’t take part, as shown in the reasons for ineligibility in Fig. 1.

There were 17 participants withdrawn from the study due to a delay in getting the app verified by Google Fit, as shown in Fig. 1. Not being verified meant that we were limited to 100 users. The process for app verification was out of the hands of the research team, and the process took longer than expected. By the time the verification was approved there would not have been a 12-week window for participants to test the app. Participants were understanding of this issue and no formal issues were raised.

Minority ethnic groups, notably Leicester’s Asian and Asian British population, were under-represented in this study - participant ethnicity breakdown is included in Table 1. The cohort’s age, sex and ethnicity influence the generalizability of research findings to other population groups, therefore validation in other cohorts may be required.

Due to slower than expected recruitment, we relaxed the criteria of the participants needing to be diagnosed with a specific LTC. While this has benefits to the study in terms of meeting the required sample size, we moved away from the original target population. Relaxing the criteria meant that we may have ended up testing the app in a more active group where the app is specifically designed for individuals who are less active. Almost a third of our users use fitness trackers on a regular basis, and are therefore likely to be more exposed to other health and fitness apps. While testing in LTC patients would have been preferred, it would be more important in an effectiveness study, less so in a usability and acceptability study. This outcome led us to conclude that perhaps in a trial setting, recruitment of participants through GP referral, rather than self would help to ensure we target the most suitable participant group.

Although not powered to detect change, we have reflected on the reasons why the RPAQ is declining over time. The weather may have affected outdoor physical activity, and as participants use the app and learn more about their levels of physical activity, they may have overestimated their level of physical activity at baseline. This highlights the need for a control group in similar studies, as the control group may have also had declining physical activity, but the difference in the two groups may be significant. This provides justification for a future two arm randomised controlled trial.

### Future work

We have gained informed consent from the P-STEP participants for the data collected in this study to be transferred to the EXCEED study team. This will enable linkage to electronic health records (EHR) and allows the EXCEED study team to analyse long term outcomes such as mortality, cardiovascular events in the future. The primary data collected in this study, together with EHR records will allow the monitoring of long-term clinical outcomes.

Finding out reasons for low interest seems important and information could be collected about this by emailing a short survey asking “we recently sent you information about a new study, please let us know why you were not interested” with some options. This was beyond the scope of this study but would have been useful. Future work to convert the app to iOS would be useful due to the number of applicates rejected for this reason. The IOS development is currently being undertaken within the research team. Finally, a randomised control trial to assess the effectiveness of the app compared with a control group is warranted.

Recruitment in digital health trials can be challenging, especially in older or clinical populations. This study highlighted difficulties in app installation, technological literacy, and seasonal timing. Future recruitment strategies might benefit from in person onboarding sessions, seasonal planning to coincide with better weather and integrating wearable devices.

## Conclusion

The purpose of this study was to assess the usability and acceptability of the P-STEP app among participants with long term conditions. The results show that the P-STEP app may be a useful tool for promoting outdoor exercise in certain patient groups. We intend to use the results from this feasibility study to make improvements to future iterations of the app and to design further research studies.

## Supplementary Information

Below is the link to the electronic supplementary material.

Supplementary Material 1.

## Data Availability

The data that support the findings of this study are available from the European Space Agency but restrictions apply to the availability of these data, which were used under license for the current study, and so are not publicly available. Data are however available from the corresponding author (hmw33@leicester.ac.uk) upon reasonable request and with permission of the European Space Agency.
