# Supplementary material for: A feasibility study evaluating the usability and acceptability of the personalised space technology exercise platform mobile application
Source: Sci Rep. 2025 Oct 17;15:36370. doi: 10.1038/s41598-025-20314-0 (PMC12534489; doi:10.1038/s41598-025-20314-0)
Supplement: Supplementary file 1 — Supplementary Material 1. [file 41598_2025_20314_MOESM1_ESM.docx]

**A feasibility study evaluating the usability and acceptability of the Personalised Space Technology Exercise Platform mobile application**

**Supplementary information**

**Figure S1: SPIRIT figure**

| **TIMEPOINT** | ***-t*** | ***0*** | ***6 week*** | ***12 week*** |
| --- | --- | --- | --- | --- |
| Registration | X |  |  |  |
| Eligibility screen | X |  |  |  |
| Enrolment | X |  |  |  |
| Informed consent | X |  |  |  |
| App deployment |  | X |  |  |
| *Baseline* | X |  |  |  |
| *6 weeks* |  |  | X |  |
| *12 weeks* |  |  |  | X |

**Table S1: Previous experience using technology**

| **Technology usage:** | **Freq (%)** |
| --- | --- |
| **Computer** |  |
| Use daily | 53 (57) |
| Used in last week | 21 (23) |
| Used in last month | 3 (3) |
| Used in last year | 5 (5) |
| Never use | 2 (2) |
| Given up using | 4 (4) |
| Missing | 5 (5) |
| **Tablet** |  |
| Use daily | 34 (37) |
| Used in last week | 8 (9) |
| Used in last month | 10 (11) |
| Used in last year | 4 (4) |
| Never use | 21 (23) |
| Given up using | 2 (2) |
| Missing | 14 (15) |
| **Smartphone** |  |
| Use daily | 91 (98) |
| Used in last week | 2 (2) |
| Used in last month | 0 |
| Used in last year | 0 |
| Never use | 0 |
| Given up using | 0 |
| Missing | 0 |
| **Fitness Device** |  |
| Use daily | 30 (32) |
| Used in last week | 3 (3) |
| Used in last month | 3 (3) |
| Used in last year | 4 (4) |
| Never use | 41 (44) |
| Given up using | 5 (5) |
| Missing | 7 (8) |
| **App (Ever used)** |  |
| Weather | 79 (85) |
| Exercise | 46 (50) |
| Health | 33 (35) |
| None | 5 (5) |
| Pollution | 0 |

**Table S2: Item responses, System Usability Scale, Bespoke Usability Scale, User Engagement Scale & general usage and feedback questions about the app**

| **System usability score responses** | **Strongly Disagree/ Disagree** | **Neither agree or disagree** | **Agree / strongly agree** |
| --- | --- | --- | --- |
| I think that I would like to use this app frequently | 26 (42%) | 4 (7%) | **31 (50%)** |
| I found this app unnecessarily complex | 28 (46%) | 14 (23%) | 19 (31%) |
| I thought this app was easy | 11 (18%) | 19 (31%) | **31 (51%)** |
| I think that I would need assistance to be able to use this app | **46 (76%)** | 6 (10%) | 9 (15%) |
| I found the various functions in this app were well integrated | 11 (18%) | 31 (51%) | 19 (31%) |
| I thought there was too much inconsistency in this app | 22 (36%) | 24 (40%) | 15 (25%) |
| I would imagine that most people would learn to use this app very quickly | 11 (18%) | 10 (16%) | **40 (66%)** |
| I found this app very awkward to use | **35 (57%)** | 15 (25%) | 11 (18%) |
| I felt very confident using this app | 11 (18%) | 11 (18%) | **39 (64%)** |
| I needed to learn a lot of things before I could get going with this app | **40 (66%)** | 12 (20%) | 9 (15%) |
| **Bespoke usability** |  |  |  |
| The app provided me with walking guidance I felt was specific to my needs | 17 (28%) | 24 (39%) | 20 (33%) |
| The app provided me with up-to-date air quality information | 4 (7%) | 5 (8%) | **52 (82%)** |
| The app motivated me to walk more | 22 (36%) | 12 (20%) | 27 (44%) |
| The app allowed me to plan my walking route | 23 (38%) | 27 (44%) | 11 (18%) |
| I feel that the app is suitable for my age group | 8 (13%) | 19 (31%) | **34 (56%)** |
| The app allowed me to track my progress | 8 (13%) | 16 (26%) | **37 (61%)** |
| The app reassured me it would keep my data protected | 1 (2%) | 28 (46%) | **32 (52%)** |
| The app allowed me to show friends and family my progress | 5 (9%) | **36 (59%)** | 20 (33%) |
| The walking guidance recommended to me matches my ability | 10 (17%) | 27 (45%) | 24 (39%) |
| **User engagement scale responses** |  |  |  |
| I lost myself in the experience of using P-STEP | **33 (54%)** | 20 (33%) | 8 (13%) |
| The time I spent using the app just slipped away | 26 (43%) | 27 (44%) | 8 (13%) |
| I was absorbed in the experience of using P-STEP | **33 (54%)** | 19 (31%) | 9 (13%) |
| I felt frustrated while using P-STEP | 27 (44%) | 11 (18%) | 23 (38%) |
| I found P-STEP confusing to use | **36 (60%)** | 14 (23%) | 11 (18%) |
| Using P-STEP was mentally and physically demanding | **42 (69%)** | 14 (23%) | 5 (8%) |
| P-STEP was attractive | 10 (16%) | **34 (56%)** | 17 (28%) |
| P-STEP was visually appealing | 8 (14%) | **33 (54%)** | 20 (33%) |
| P-STEP appealed to my senses | 16 (27%) | 30 (49%) | 15 (25%) |
| Using P-STEP was worthwhile | 17 (28%) | 14 (23%) | 30 (49%) |
| My experience of using P-STEP was rewarding | 21 (35%) | 15 (25%) | 25 (41%) |
| I felt interested in the experience of using P-STEP | 14 (22%) | 10 (16%) | **37 (61%)** |
| General usage and feedback |  |  |  |
| In the last 6-weeks, how many times a week did you use this app?  0  1-3  4-6  7+ | - | 5 (7%)  33 (46%)  24 (33%)  10 (14%) | 11 (18%)  20 (33%)  20 (33%)  10 (16%) |
| Would you use this app again in the future?  Yes  No  Unsure | - | 31 (43%)  28 (39%)  13 (18%) | 29 (48%)  19 (31%)  13 (21%) |
| How likely are you to recommend this app to a friend? (0-10) | - | 4.9 (3.3) | 5.2 (3.6) |
| Please give this app an overall rating (1-5) | - | 3.1 (1.2) | 3.1 (1.4) |
| **Self-perceived walking pace** |  |  |  |
| Do you think your walking pace has changed as a result of using the P-STEP app?  Yes  No  Missing | - | 17 (24%)  55 (76%) | 14 (23%)  47 (77%) |
| If yes, how would you now describe your usual walking pace?  Slower pace - less than 3 miles per hour  Steady average pace - between 3 and 4 miles an hour  Brisk pace - more than 4 miles an hour  Missing | - | 2  13  2 | 3  7  2  2 |

| **Table S3: Non-routine GP visits and unexpected hospitalisations** |  |  |
| --- | --- | --- |
|  | **6 weeks** | **12 weeks** |
| A non-routine GP appointment due to your chronic condition(s)? | 4 | 4 |
| A non-routine GP appointment due to anything other than your chronic condition(s)? | 17 | 7 |
| Hospitalized as a result of your chronic condition(s)? | 0 | 0 |
| Hospitalized for anything other than your chronic condition(s)? | 1 | 0 |

| **Table S4: Subgroup analyses** | | | | |
| --- | --- | --- | --- | --- |
| **Usability scores by disease group** | | | | |
|  | Disease = 0 | Disease = 1 | Diff (95% CI) | p |
| N (6 week) | 51 | 21 |  |  |
| N (12 week | 44 | 17 |  |  |
|  |  |  |  |  |
| SUS (6 week) | 58.45 (18.94) | 56.67 (23.13) | 1.783 (-8.72 to 12.29) | 0.736 |
| SUS (12 week) | 63.52 (22.55) | 56.91 (20.74) | 6.611 (-6.00 to 19.23) | 0.299 |
| Bespoke (6 week) | 65.70 (14.69) | 65.4 (16.53) | 0.3112 (-7.57 to 8.19) | 0.938 |
| Bespoke (12 week) | 66.30 (14.86) | 68.11 (14.82) | -1.780 (-10.32 to 6.72) | 0.674 |
| UES (6 week) | 3.08 (0.742) | 3 (0.940) | 0.084 (-0.33 to 0.50) | 0.688 |
| UES (12 week) | 3.08 (0.783) | 3.10 (0.823) | -0.027 (-0.48 to 0.43) | 0.905 |
|  |  |  |  |  |
| **Usability scores by experience using fitness tracker** | | | | |
|  | Fitness tracker = 0 | Fitness tracker = 1 | Diff (95% CI) | p |
| N (6 week) | 49 | 22 |  |  |
| N (12 week | 42 | 19 |  |  |
|  |  |  |  |  |
| SUS (6 week) | 58.47 (20.38) | 56.70 (19.95) | 1.765 (-8.60 to 12.13) | 0.735 |
| SUS (12 week) | 61.96 (21.79) | 61.05 (23.32) | 0.912 (-11.41 to 13.24) | 0.883 |
| Bespoke (6 week) | 66.26 (15.66) | 64.25 (14.20) | 2.007 (-5.66 to 9.68) | 0.603 |
| Bespoke (12 week) | 68.89 (14.77) | 62.34 (14.02) | 6.550 (-1.53 to 14.63) | 0.110 |
| UES (6 week) | 3.08 (0.83) | 3.01 (0.73) | 0.067 (-0.34 to 0.47) | 0.748 |
| UES (12 week) | 3.14 (0.86) | 2.96 (0.60) | 0.180 (-0.26 to 0.62) | 0.416 |
|  |  |  |  |  |
| **Usability scores by non-usage** | | | | |
|  | Non users not removed | Non users removed | Diff (95% CI) | p |
| N (6 week) | 71 | 67 |  |  |
| N (12 week | 61 | 58 |  |  |
|  |  |  |  |  |
| SUS (6 week) | 57.92 (20.12) | 59.40 (18.97) | 1.48 (-5.11 to 8.07) | 0.658 |
| SUS (12 week) | 61.69 (22.09) | 62.41 (22.29) | 0.733 (-7.32 to 8.79) | 0.857 |
| Bespoke (6 week) | 65.62 (15.14) | 66.50 (14.41) | 0.886 (-4.06 to 5.83) | 0.724 |
| Bespoke (12 week) | 66.81 (14.75) | 67.06 (15.08) | 0.242 (-5.22 to 5.70) | 0.930 |
| UES (6 week) | 3.056 (0.79) | 3.12 (0.76) | 0.061 (-0.20 to 0.32) | 0.645 |
| UES (12 week) | 3.083 (0.79) | 3.12 (0.79) | 0.039 (-0.25 to 0.33) | 0.787 |

**Table S5: Free text responses**

|  |  |  |
| --- | --- | --- |
| **Please enter any suggestions to improve the app?** | | **Please enter any other feedback you have** |
| *“Make it useable and relevant.”*  *“I wasn't really sure what I was supposed to get from the app.”* | | *“Did not understand what it was all for.”* |
| *“Rather than held it throughout the walk, which was sometimes inconvenient and also meant my phone got wet.”*  *“Was not clear if I was expected to complete longer individual walks, rather than the shorter walks that I was doing.”*  *“Not clear if AQ relates only to environmental pollution levels. My asthma is provoked by plant spores, pollen, mould and is particularly problematic in summer and in extreme heat. Pollution from the environment, such as transport fumes, does not appear to affect it. The more problematic walk for me would be walking in countryside in summer. (I would then check pollen levels although my breathing would normally tell me quite quickly). Therefore, is the AQ advice on the app relevant to me?”*  *“I was confused by the star system for feedback on the walks. I would have preferred 0-10 system”*  *“When trying to complete the star feedback the app did not always let me click on the stars I wanted. I therefore gave up.”* | | *“The app may be more useful if walking was my only form of exercise, although the AQ rating remains a problem for me. I would be interested to see what the AQ shows in the summer months.”*  *“An enjoyable walk for me is strolling with a friend which I realise is not brisk but it is movement, albeit gentle, and is of benefit to my mental health.”*  *“I don't think I need a reward system. The only reward I need is staying healthy and active despite the limitations of variable health and old age.”*  “*Overall, I have enjoyed trying out the app but do not think it is useful to me at this stage in my life. Thank you for your very quick responses when I had questions.”* |
| *“Maybe a quick record walk button rather than the multistep process.”* | |  |
| *“Cut down the number of screen touches needed to start a walk”* | | *“Too many notifications”* |
| *“Automatically recognise you're walking”* | |  |
|  | | *“Several times it lost my walk information before I was able to log it.”* |
| *“My watch knows when I am walking and driving.*  *Integrate PStep with this function to make it automatic”* | |  |
| *“Could be a made a bit clearer when trying to find information on this app”* | | *“The weather has limited my use of this app as I have asthma it’s difficult to walk a lot in the cold and wet think, I would be able to use it more in better weather”* |
| *“Couldn't always down load walk as no WIFI available. Could it be done through data”* | | *“Just what I needed. Can I continue using the app no 12 weeks have finished?”* |
| *“Forgot to start a walk more often than remembered so the fact that it's not automatically recording like a Fitbit say is a drawback”* | |  |
| *“Instructions to use the app”*  *“Easier to start recording a walk”*  *“Cut off if stopped moving”* | |  |
| *“Perhaps make the writing in the app easier to understand”* | | *“It is easy to use”*  *“The air quality index is a good idea”* |
| *“I was warned by play store that it was unsecure to use and kept getting warnings that my Google account was being compromised so I deleted the app”* | |  |
| *“Automatically turn on”* | |  |
|  | | *“The app did not seem to work my end so not sure if data was collected your end.”* |
| *“Air quality is useful to know”*  *“I'm not sure that the app is as motivating as other technology like Fitbit”* | | *“Not sure the distance recorded is accurate”* |
|  | | *“I like the app”* |
|  | | “*Your App makes me realise that I need to walk more!! So glad I’m doing this research”*  *“My walk routes are mainly getting to work.”*  *“I normally walk in Lake district/Wales and would like to use this app but weather has been awful. I'm hoping the weather will improve for me to get out of the city and see how the app works then.”* |
|  | | *“Probably useful for someone who goes walking for pleasure rather than to get somewhere”* |
| *“I would like to be able to repeat a walk instead of having to rename the same walk.”* | | *“I have enjoyed using the app it has encouraged me to commit to meeting or exceeding the target.”* |
| “*In my area of Birstall, it didn't suggest routes for a gentle walk. I know this is developmental, but that would have been very useful and interesting.”*  *“I use a WALKER and I realise that the type of walk is limited.”*  *“As you ask about health, perhaps it could indicate where public defibrillators are located en route.”* | | *“Yes, you have a ...start, stop button. why not a pause button when you stop for a break in the route and then you can restart it. it would give you more accurate data.”* |
| *“The app is not comparable to Strava (free version), which I think your app should use as a basis.”*  *“It needs to auto-pause”* | | *“This app needs to tell user to switch battery saver off to improve tracking.”* |
| *“I don't have any serious health problems. I don't think it has much to offer for those without a relevant health problem, even older people.”* | | *“P step was tricky to use at first but straightforward once I got it working.”*  *“I had never used google fit but after downloading it for this project, I check it quite often. Google fit is enough for me.”*  *“I'm sure it would be helpful for those with chronic problems seeking extra motivation”* |
| *“An edit button for altering the time taken to walk; I did a walk on one occasion but forgot to press stop once the walk had ended, so wasn't able to record accurately as the walk would have been 2 hours over what actually done.“* | | *“I really enjoyed using this app. My usage was less in this period of time due to icier conditions outside which I won't walk on, and also, I had a very bad sinus infection for 4 weeks which also impacted on how much I felt like doing. Really hope the app becomes available for use.”* |
| *“Stop resetting the time every Monday.”*  *“Enable long press on the mins setting to speed through the display”* | | *“Main problem is that it is phone based and so is not viewable during a walk as the phone is in my pocket as opposed to a wrist-based app that can easily be viewed at any time.”*  *“I also found it odd for the app to be based on time rather spent walking rather that the number of steps taken.”* |
| *“Auto detect when walking starts, pauses and stops.”*  *“I got fed up of having to stop and start or pause and eventually forgot to stop it until i realised after half an hour of driving.”*  *“There are good apps out there which are already doing this but yours has the air quality, just needs to improve on the functionality.”* | | *“It’s been raining heavily, not ideal conditions for walking. the study needs to be repeated when the weather is more conducive to walking.”* |
| *“Automatically detect that you are walking”* | |  |
| *“Make sure it works as Strava does”* | |  |
|  | | *“On the whole the app is good.”* |
| *“I never got it to work properly. It was easier to just use my fitness watch to monitor my activity.”* | | *“I liked the air quality information.”*  *“The simplicity might appeal to people who were not very tech-literate.”*  *“It also could be useful to motivate people who were very inactive.”* |
| *“The star system for how challenging difficult is confusing. I have put 2 stars as the walk was tedious and easy but it looks as though I should have put four or five as two stars looks as though it means challenging. I think that "easy" should be on the left-hand side.”* | | *“I changed the target set very quickly and have since increased it - and am still exceeding it. I walked far more as I was trialling the app and this has now become a habit.”*  *“The enjoyment etc is not relevant as I am trudging the local streets most of the time which is not enjoyable!”* |
| *“It would be useful to be able to use the app when using the treadmill/bike/rower at the leisure centre where I went when the weather was bad.”* | | *“I really got into using the app, using it to measure improvement in pace and the length of time spent walking. Really motivated me to get out and walk. I lost weight, increased my speed and felt fitter.”* |
| *“Make it auto detect when walking, I’m sure you could show the route taken using gps”* | | *“I forgot to use most times when went out”* |
| *“If the app could automatically pause when you stop walking rather than having to remember to pause it.”* | |  |
| *“Need to be able to access the app offline”*  *“Not helpful that you have to 'start' the walk and stop it”*  *“On occasions I have started the walk, the air quality popped up and I wrongly did not start the walk. On another occasion the walk didn't appear to be recorded, which is frustrating. It would be better if all walks were recorded like fitbit, e.g. I was walking a lot around the house, garden, up and down stairs etc which obviously were never recorded and add up considerably.”* | | *“I don't have a lot of data so it would be a much more useful tool if walks were recorded without the need to switch on mobile data at a cost.”* |
| *“A lot of good stuff but improvements.”*  *“Allow exercise bikes extra”*  *“Sometimes forgot to switch the app off, got in the car and it was counted as part of the walk, a method to stop the walk.”* | | *“A useful tool.”* |
| *“Make it easier to install”* | | *“Couldn't use the app as despite instructions I couldn't install it as for some reason Google blocked it. Couldn't go back or forward from the page at the time Google blocked. So uninstalled it and decided not worth the hassle.”* |
| *“Make it retain your weekly target rather than needing to reset every week. (Although resetting does tend to make you increase your target. Did for me anyway!)”* | |  |
| *“Make it compatible with smart devices, such as Garmin”* | |  |
| *“Give reminders to use it.“* | | *“I don't remember to use it. I couldn't use it on holiday, although I did a lot of walking, as my phone contract wouldn't work in the country I visited.”* |
| *“App is ok but just not for me.”* | | *“I kept forgetting to start & finish the recording, so data was inaccurate and useless.”* |
| *“Did have a few problems with the app over recent weeks, reported to the helpdesk.”* | | *“Needed to remember to "log out" as I felt the app ate my battery.”*  *“Liked the mph and elevation interesting.”* |
| *“App should pause automatically when no walking detected.”*  *“I discarded some walks after forgetting to stop the app at the end of the walk.”*  *“Map display area should be larger and more manageable.”* | | *“The app is motivational but unsatisfactory.”* |
| *“To get to start you have to click through a few things, could be reduced”* | | *“Need to try it more”* |
| *“It would be useful if the app automatically paused when I stop walking.”* | | *“Useful app for air quality, temperature and accurate walks.”* |
| *“One button to start”*  *“Automatic suspend when not walking for 5 minutes”* | |  |
| *“If possible, it would be good if the app was programmed to pause or stop after say about 10 minutes of non-activity as a few times I forgot to switch it off.”*  *“Vibrate or ring when it pauses so you know to switch it back on.”*  *“A reminder each day to remember to switch it on when moving would be good as sometimes I forgot that too!!”* | | *“I thought it was a nice little app which would appeal to people who have not used one before but wouldn't be sophisticated enough for the more serious sportsperson.”*  *“I feel, though, that this is aimed at getting people who have not done much exercise and need something a little simpler to start with.”*  *“I was particularly impressed with the GPS and how accurate it was and would definitely use it again.”*  *“I've given it a 3 rating because I would like it to pause or stop after a certain amount of inactivity, to vibrate when it does and to give reminders to get moving.”* |
